# Apelin Ameliorates TNF-α-Induced Reduction of Glycogen Synthesis in the Hepatocytes through G Protein-Coupled Receptor APJ

**DOI:** 10.1371/journal.pone.0057231

**Published:** 2013-02-21

**Authors:** Jiaojiao Chu, Hangxiang Zhang, Xiuqing Huang, Yajun Lin, Tao Shen, Beidong Chen, Yong Man, Shu Wang, Jian Li

**Affiliations:** 1 Key Laboratory of Geriatrics, Beijing Institute of Geriatrics & Beijing Hospital, Ministry of Health, Beijing, China; 2 Department of Gerontology, Xi-Jing Hospital, Fourth Military Medical University, Xian, China; 3 Zhejiang Hospital, Hangzhou, Zhejiang Province, China; Universita Magna-Graecia di Catanzaro, Italy

## Abstract

Apelin, a novel adipokine, is the specific endogenous ligand of G protein-coupled receptor APJ. Consistent with its putative role as an adipokine, apelin has been linked to states of insulin resistance. However, the function of apelin in hepatic insulin resistance, a vital part of insulin resistance, and its underlying mechanisms still remains unclear. Here we define the impacts of apelin on TNF-α-induced reduction of glycogen synthesis in the hepatocytes. Our studies indicate that apelin reversed TNF-α-induced reduction of glycogen synthesis in HepG2 cells, mouse primary hepatocytes and liver tissues of C57BL/6J mice by improving JNK-IRS1-AKT-GSK pathway. Moreover, Western blot revealed that APJ, but not apelin, expressed in the hepatocytes and liver tissues of mice. We found that F13A, a competitive antagonist for G protein-coupled receptor APJ, suppressed the effects of apelin on TNF-α-induced reduction of glycogen synthesis in the hepatocytes, suggesting APJ is involved in the function of apelin. In conclusion, we show novel evidence suggesting that apelin ameliorates TNF-α-induced reduction of glycogen synthesis in the hepatocytes through G protein-coupled receptor APJ. Apelin appears as a beneficial adipokine with anti-insulin resistance properties, and thus as a promising therapeutic target in metabolic disorders.

## Introduction

Insulin resistance, defined as a diminished ability of cells such as adipocytes, skeletal muscle cells and hepatocytes to respond to the action of insulin, is not only the pathophysiological hallmark of type 2 diabetes and the metabolic syndrome [1], but also an independently and strongly associated factor with an increased risk of coronary disease [2], [3], heart failure [4] and mortality [5].

TNF-α has been implicated in the pathogenesis of insulin resistance in vitro and in vivo [6]. Elevated plasma TNF-α levels may play an important role in insulin resistance by impairing insulin signaling [7]. Moreover, our previous study indicated that in cultured human HepG2 hepatocytes, TNF-α induced insulin-resistance, as assessed by their decreased capacity to accumulate glycogen in the presence of insulin [8].

Adipose tissue has been considered as a major endocrine organ producing several adipokines affecting insulin resistance [9]. Apelin, a novel adipokine, is the specific endogenous ligand of G protein-coupled receptor APJ [10]. The human apelin gene that is located on chromosomeXq25-26 expresses a 77-amino acid prepropeptide that is subsequently cleaved post-translationally into several active forms, including apelin-36, apelin-17, apelin-13, apelin-12, which are all agonists of apelin receptor [11], [12]. Apelin has gained increasingly attention in recent years, for it appears to have numerous distinct biological activities in a variety of organs [13]. Consistent with its putative role as an adipokine, apelin has been linked to states of insulin resistance. Apelin expression was up-regulated by insulin in the adipose tissue [10], while in the pancreas, apelin could decrease insulin secretion [14]. Moreover, it has been proved that apelin is necessary for the maintenance of insulin sensitivity [15]. Interestingly, glucose utilization in the muscle and adipose tissue could be stimulated by apelin, and insulin sensitivity would be increased subsequently [16]. However, the function of apelin in hepatic insulin resistance, a vital part of insulin resistance, and its underlying mechanisms still remains unclear. Here we studied the impacts of apelin on TNF-α-induced reduction of glycogen synthesis in the hepatocytes. We show novel evidence suggesting that apelin ameliorates TNF-α-induced reduction of glycogen synthesis in the hepatocytes through G protein-coupled receptor APJ. Apelin appears as a beneficial adipokine with anti-insulin resistance properties, and thus as a promising therapeutic target in metabolic disorders.

## Results

### Apelin reverses TNF-α-induced reduction of glycogen synthesis in HepG2 hepatocytes and mouse primary hepatocytes

**Figure 1:**
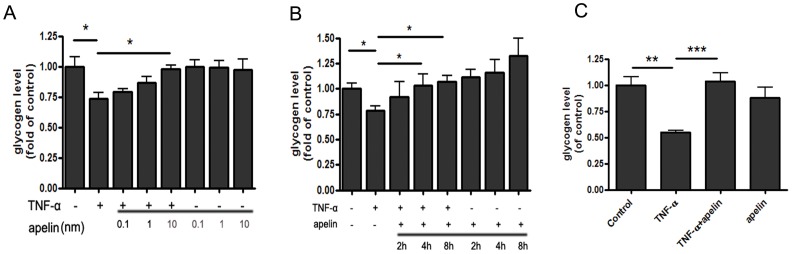
Apelin reverses TNF-α-induced reduction of glycogen synthesis in HepG2 hepatocytes and mouse primary hepatocytes. Exposure of HepG2 hepatocytes to apelin-13 (0.1, 1, 10 nmol/L) before treatment with 10 ng/ml TNF-α for 24 h elevated glycogen content in a dose- and time-dependent manner (A and B). The treatment of 10 nmol/L apelin-13 for 4 h reversed TNF-α-induced reduction of glycogen synthesis in mouse primary hepatocytes (C). Data represent the means ± S.E.M., n = 3 independent experiments. * p<0.05; ** p<0.01 and *** p<0.001 by ANOVA test (v.s. control or TNF-α).

To observe effects of apelin on glycogen synthesis in the hepatocytes, human HepG2 hepatocytes were treated with 10 ng/ml TNF-α for 24 h to reduce intracellular glycogen synthesis. Then, HepG2 cells were treated with different concentrations of apelin 13 (0.1, 1, 10 nmol/L; Phoenix Pharmaceuticals, USA), followed by exposure to 10 ng/ml TNF-α for 24 h. The results indicate that glycogen content was dose-dependently increased by treatment of HepG2 cells with apelin (Fig. 1A). Next, HepG2 cells were treated with 10 nmol/L apelin for different time (2, 4, 8 h). As shown in Fig. 1B, apelin led to a time-dependently elevated glycogen content of HepG2 cells. Therefore, in the following experiments, HepG2 cells were treated with 10 nmol/L apelin for 4 h, followed by incubation 10 ng/ml TNF-α for 24 h. We also quantified cell viability in HepG2 cells treated with 10 nmol/L of apelin for 4 h and 10 ng/ml TNF-α for 24 h by a 3-(4,5-dimethylthiazol-2-yl)-2,5-diphenyltetrazoliumbromide assay to exclude the side effects associated with apelin and TNF-α, such as apoptosis. The results indicate that no cytotoxicity was seen in connection with the exposure of HepG2 cells to apelin (10 nmol/L, 4 h) and TNF-α (10 ng/ml, 24 h)(data not shown). Moreover, the glycogen content was reduced in mouse primary hepatocytes treated with 10 ng/ml TNF-α for 24 h. However, treatment of 10 nmol/L apelin impaired the effect of TNF-α on glycogen synthesis in mouse primary hepatocytes (Fig. 1C). Taken together, these results indicate that apelin could reverse TNF-α-induced reduction of glycogen synthesis.

### Apelin improves insulin signaling pathway in the hepatocytes treated by TNF-α

**Figure 2:**
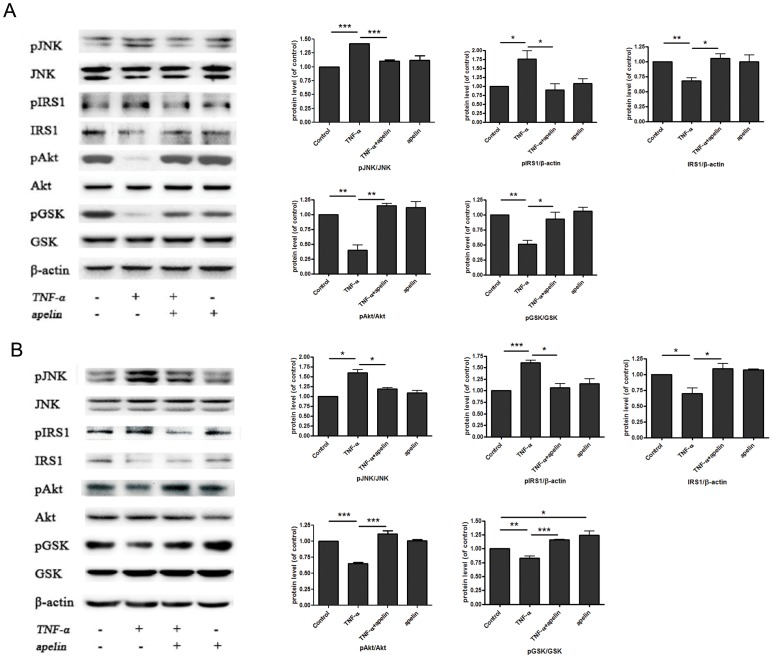
Apelin improves insulin signaling pathway in the hepatocytes treated by TNF-α. Apelin-13 improved insulin signaling pathway (JNK-IRS1-AKT-GSK) in HepG2 cells (A) and mouse primary hepatocytes (B) treated with 10 ng/ml TNF-α for 24 h. Data represent the means ± S.E.M., n = 3 independent experiments. * p<0.05; ** p<0.01 and *** p<0.001 by ANOVA test (v.s. control or TNF-α).

Since insulin signaling pathway plays an important role in glycogen synthesis, we then investigated whether and how apelin improved insulin signaling pathway in the hepatocytes treated by TNF-α.As shown in Fig. 2A, JNK was activated in response to TNF-α treatment in HepG2 cells. In parallel with increased phosphorylation of JNK, phosphorylation of the residue Ser307 in IRS-1, accompanied by reduced IRS-1 levels was stimulated by TNF-α treatment. Moreover, TNF-α-induced activation of JNK led to impaired phosphorylation of AKT and GSK. However, these changes of JNK, IRS-1, AKT and GSK induced by TNF-α were reversed via apelin treatment. The effects of apelin on TNF-α-induced impaired insulin signaling pathway were further assessed in mouse primary hepatocytes (Fig. 2B). These observations suggest that apelin ameliorates TNF-α-induced reduction of glycogen synthesis in the hepatocytes by improving insulin signaling pathway.

### Injection of apelin increases glycogen level and improves insulin signaling pathway in the liver tissues of TNF-α-treated C57BL/6J mice

**Figure 3:**
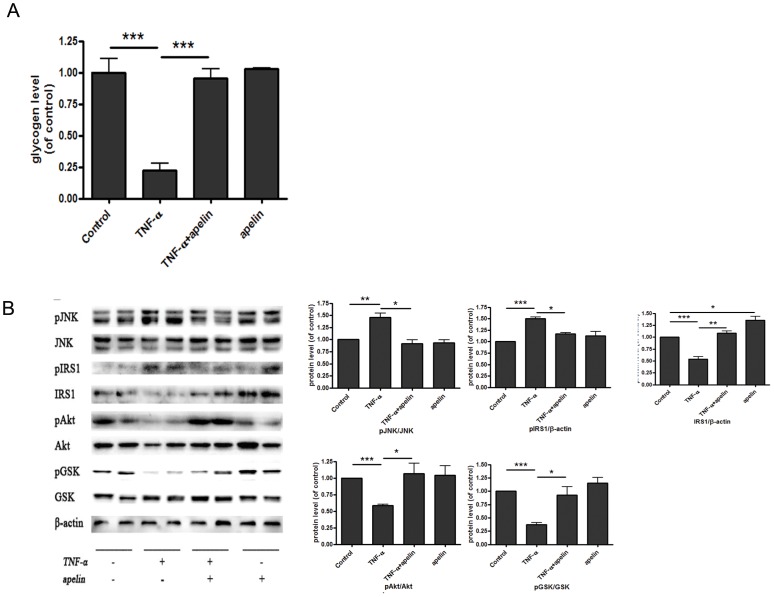
Injection of apelin increases glycogen level and improves insulin signaling pathway in liver tissues of TNF-α-treated C57BL/6J mice. An intraperitoneal injection of 20 nmol/kg apelin-13 led to increased glycogen level in liver tissues of C57BL/6J mice treated by TNF-α (A). Moreover, insulin signaling pathway was impaired in liver tissues treated with TNF-α. These changes of JNK, IRS-1, AKT and GSK induced by TNF-α were reversed via apelin injection (B). Data represent the means ± S.E.M., n = 5 mice per group. * p<0.05; ** p<0.01 and *** p<0.001 by ANOVA test (v.s. control or TNF-α).

To further assess the effects of apelin on glycogen synthesis in vivo, 12-week-old male C57BL/6J mice were injected with 7.01 µg/ml TNF-α by pumps for 7 days and the livers of mice were collected. We found a decrease of glycogen level in the liver tissues of TNF-α-treated C57BL/6J mice. However, an intraperitoneal injection of 20 nmol/kg apelin-13 led to increased glycogen level in the liver tissues of C57BL/6J mice treated by TNF-α (Fig. 3A). Moreover, as shown in Fig. 3B, insulin signaling pathway was impaired in the liver tissues treated with TNF-α. These changes of JNK, IRS-1, AKT and GSK induced by TNF-α were reversed via apelin injection.

### Apelin affects TNF-α-induced reduction of glycogen synthesis in the hepatocytes through G protein-coupled receptor APJ

**Figure 4:**
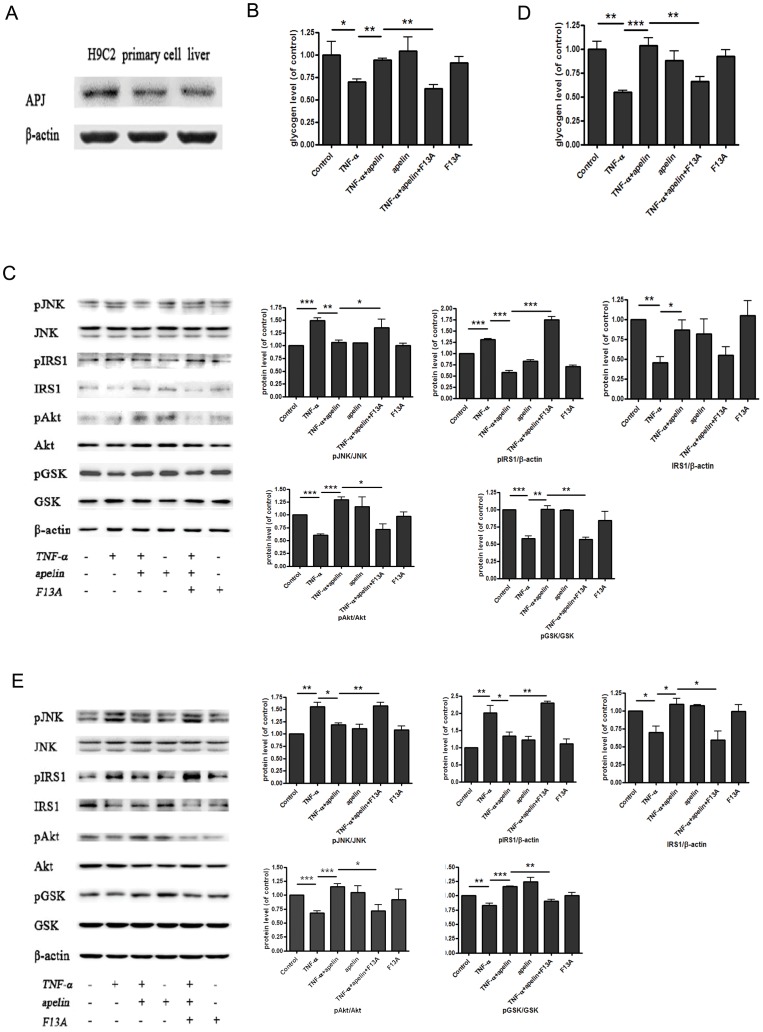
Apelin affects TNF-α-induced reduction of glycogen synthesis in the hepatocytes through G protein-coupled receptor APJ. APJ expressed in HepG2 cells, primary mouse hepatocytes and liver tissues of mice (A). 20 nmol/L F13A, a competitive antagonist for APJ, was exposed to HepG2 cells and mouse primary hepatocytes treated with TNF-α or/and apelin. The regulation of apelin in glycogen synthesis and insulin signaling pathway was inhibited by treatment of F13A in HepG2 cells (B and C) and mouse primary hepatocytes (D and E). Data represent the means ± S.E.M., n = 3 independent experiments. * p<0.05; ** p<0.01 and *** p<0.001 by ANOVA test.

G protein-coupled receptor APJ has been known to be the unique receptor of apelin. In order to elucidate whether APJ is involved in the function of apelin in glycogen synthesis, we measured the expression of APJ in HepG2 cells, mouse primary hepatocytes and liver tissues of mice by Western blot. As shown in Fig. 4A, APJ receptor can be detected in HepG2 cells, mouse primary hepatocytes, as well as liver tissues of mice. F13A is an established antagonist of APJ receptor with a substitution of phenylalanine by alanine in the C-terminal of apelin-13. [17]. Therefore, to further assess the role of apelin in the maintenance of insulin sensitivity, 20 nmol/L F13A (Phoenix Pharmaceuticals, USA) was exposed to HepG2 cells and mouse primary hepatocytes treated with TNF-α or/and apelin. The results reveal that regulation of apelin in glycogen synthesis and insulin signaling pathway was inhibited by treatment of F13A in HepG2 cells (Fig. 4B, C). These changes are consistent with data from mouse primary hepatocytes (Fig. 4D, E).

### Injection of F13A, a competitive antagonist for APJ, suppresses the effects of apelin on glycogen synthesis and insulin signaling pathway in TNF-α-treated mice

**Figure 5:**
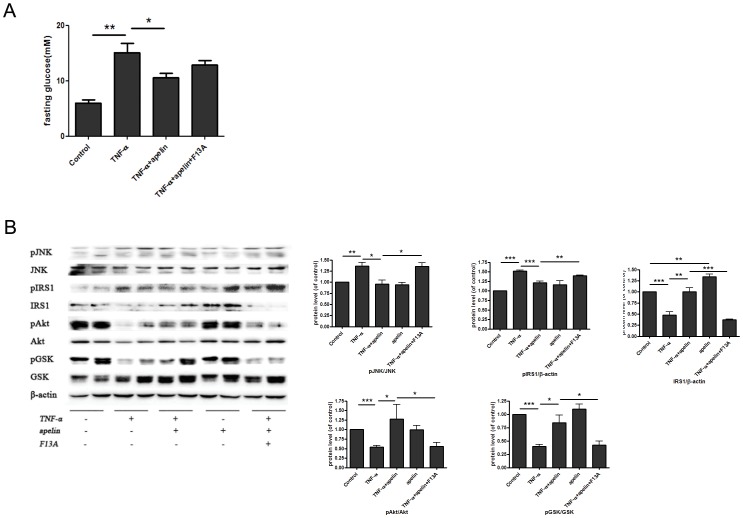
Injection of F13A suppresses the effects of apelin on glycogen synthesis and insulin signaling pathway in TNF-α-treated mice. F13A (20 µg/mouse) or/and apelin 13 (20 nmol/kg) was injected in TNF-α-treated C57BL/6J mice. Injection of F13A suppressed the effects of apelin on glycogen synthesis (A) and insulin signaling pathway (B) in TNF-α-treated mice. Data represent the means ± S.E.M., n = 5 mice per group. * p<0.05; ** p<0.01 and *** p<0.001 by ANOVA test.

Finally, we injected F13A (a competitive antagonist for APJ, 20 µg/mouse) or/and apelin 13 (20 nmol/kg) in TNF-α-treated C57BL/6J mice. Interestingly, injection of F13A suppressed the effects of apelin on glycogen synthesis and insulin signaling pathway in TNF-α-treated mice (Fig. 5A, B). Taken together, our results suggest that apelin ameliorates TNF-α-induced reduction of hepatic glycogen synthesis through G protein-coupled receptor APJ.

## Discussion

In the present study, we found that (i) apelin can stimulate insulin signaling pathway and improve glycogen synthesis in TNF-α-treated hepatocytes and liver tissues of mice; (ii) APJ, the only known receptor for apelin, but not apelin, expressed in HepG2 cells, mouse primary hepatocytes and liver tissues of mice; and (iii) apelin ameliorates TNF-α-induced reduction of glycogen synthesis in the hepatocytes through G protein-coupled receptor APJ.

In recent years, apelin has been linked to states of insulin resistance. In clinical studies, it has been reported that the levels of plasma apelin were elevated in insulin-resistant subjects [18] and in morbidly obese individuals with type 2 diabetes [19], [20], compared with normal controls. However, several recent studies have shown decreased plasma apelin concentrations in newly diagnosed and untreated patients with type 2 diabetes [21], [22]. These results could be consistent with the fact that after 14 weeks of anti-diabetic treatment (rosiglitazone and metformin), plasma apelin concentrations were enhanced and the glycemic profile improved [23]. Although it is difficult to reconcile these divergent findings, the data raise the possibility of alternative regulatory pathways for apelin production in the setting of insulin resistance [24]. Moreover, it has been found that changes in plasma apelin concentrations were correlated significantly with plasma triglycerides, glucose, TNF-α, HOMA-IR and HbA1c [25]. It is known that insulin resistance occurs in certain tissues, such as adipose tissue, muscle and liver. But liver becomes resistant first, followed by muscle and, last, adipose tissue. Insulin resistance in the livers led to impaired glycogen synthesis and failure to suppress glucose production [26]. Positive correlation between the levels of plasma apelin and insulin has been observed in wild type mice, heterozygous db/+ mice and homozygous db/db mice [10]. It has been reported that apelin could stimulate glucose utilization in skeletal muscle and adipose tissue in normal and insulin-resistant mice [16]. Direct administration of apelin in preclinical animal models resulted in improved insulin sensitivity [16], [27]. Moreover, the mice with a generalized deficiency of apelin have abnormal insulin tolerance and insulin sensitivity, however, administration of exogenous apelin to these mice reversed these abnormalities [15]. These observations indicate that apelin directly increases insulin sensitivity and suggest that the elevations in circulating apelin concentrations observed in states of insulin resistance are compensatory [24]. In our study, human HepG2 hepatocytes were treated with 10 ng/ml TNF-α for 24 h to decrease intracellular glycogen synthesis. The results indicate that glycogen content was dose-dependently increased by treatment of HepG2 cells with apelin. Moreover, treatment of 10 nmol/L apelin impaired the effect of TNF-α on glycogen synthesis in mouse primary hepatocytes. Similarly, an intraperitoneal injection of 20 nmol/kg apelin 13 led to increased glycogen levels in the liver tissues of TNF-α-treated C57BL/6J mice. Taken together, these results suggest that apelin can reverse TNF-α-induced reduction of glycogen synthesis.

It has been reported that apelin is secreted predominantly from endothelial cells [28]. More recently, apelin has been found to be present in adipocytes [10], [29]. To date, evidence suggests that apelin is expressed in CNS and a range of peripheral rat tissues, including heart, liver, kidney, testis, ovary, and adipose tissue, with highest levels in the lung and the mammary gland [30]. However, we found that no apelin is expressed in HepG2 cells and mouse primary hepatocytes (data not shown). This corroborates previous report [30]. Therefore, in our experiments, exogenous apelin 13 was used to treat HepG2 and mouse primary hepatocytes, and administrate C57BL/6J mice.

Insulin signaling pathway plays a critical role in regulation of glycogen synthesis. There is strong evidence for oxidative stress-dependent changes in intracellular signaling, resulting in insulin resistance in vivo [31]. From a mechanistic perspective, increased reactive oxygen species (ROS) can trigger activation of stress-sensitive serine/threonine kinase signaling pathways, such as JNK, that, in turn, phosphorylate multiple targets, including the insulin receptor and IRS proteins [32]. Increased serine phosphorylation of IRS-1 reduces its ability to undergo tyrosine phosphorylation and may accelerate the degradation of IRS-1, followed by reduced AKT phosphorylation. It is demonstrated that AKT phosphorylated and inhibited GSK-3, followed by suppressed glycogen synthase activity, resulting in decreased glycogen synthesis. We found that ROS levels were increased in HepG2 cells by exposure to TNF-α. However, apelin significantly inhibited the generation of ROS in response to TNF-α (data not shown). It has been reported that through increased apelin production, RAS blockers could prevent the generation of ROS in differentiating adipocytes [33]. Inhibition of ROS production by apelin has also been shown in other cell types [34], [35]. Our results also show that in parallel with increased phosphorylation of JNK, phosphorylation of the residue Ser307 in IRS-1, accompanied by reduced IRS-1 levels was stimulated by TNF-α treatment in HepG2 cells. Moreover, TNF-α-induced activation of JNK led to impaired phosphorylation of AKT and GSK. However, these changes of JNK, IRS-1, AKT and GSK induced by TNF-α were reversed via apelin treatment. The effects of apelin on TNF-α-induced impaired insulin signaling pathway were further assessed in mouse primary hepatocytes and liver tissues of C57BL/6J mice.

It is consider that apelin is the specific ligand of G protein-coupled receptor APJ. Up to date, there is no evidence for multiple receptor subtypes of APJ [36], [37]. No apelin expression can be detected in liver cells despite of the general distribution in other tissues [30], [38]. As for APJ, opinions vary about whether APJ is expressed in hepatocytes [16], [30], [38]. In the present study, we measured the expression of APJ in HepG2 cells, mouse primary hepatocytes and liver tissues of mice by Western blot. The results indicate that APJ expressed in HepG2 cells, mouse primary hepatocytes and liver tissues of mice. As apelin exists in blood, we consider that apelin could enter the liver with blood-steam, activate receptor APJ in the cellular membrane of hepatocytes, and then stimulate down-stream signal molecule. Notably, F13A could competitively bind to APJ which blocking the activation the receptor by apelin. [39] Therefore, F13A is used as a competitive antagonist for APJ.In this study, 20 nmol/L F13A was exposed to HepG2 cells and mouse primary hepatocytes treated with TNF-α or/and apelin. The results reveal that regulation of apelin in glycogen synthesis and insulin signaling pathway was inhibited by treatment of F13A in HepG2 cells and mouse primary hepatocytes. These changes are consistent with data from in TNF-α-treated C57BL/6J mice.

In previous study, 10 C57BL/6J mice matched for age, body weight and glucose level were enrolled in plasma insulin level measurement by an ELISA kit (Linco) according to the manufacturer's protocol. These mice were separated into 2 groups randomly. The plasma insulin levels of mice in these 2 groups were 2.82±0.90 ng/ml and 3.00±1.71 ng/ml respectively (p = 0.85), suggesting that insulin levels were comparable among the different groups. Therefore, the plasma insulin concentration was not measured at the beginning of the treatment in the present study. It is a limitation in the present study.

In summary, this study provides novel evidence suggesting that apelin ameliorates TNF-α-induced reduction of glycogen synthesis in the hepatocytes through G protein-coupled receptor APJ. Apelin appears as a beneficial adipokine with anti-insulin resistance properties, and thus as a promising therapeutic target in metabolic disorders. However, further studies are needed to determine what the downstream signaling pathway of AJP is and how AJP signaling pathway is connected to insulin signaling pathway.

## Materials and Methods

### Animals

12-week-old male C57BL/6J mice were provided from Peking University Health Science Center. The mice matched for age, body weight and glucose level were separated for 5 groups (control, TNF-α, apelin, TNF-α+ apelin, TNF-α+apelin+F13A) with 5 mice per group and fed a standard laboratory diet in a temperature-controlled (20–24°C) and humidity-controlled (45–55%) environment. A 12 h/12 h light/dark cycle was maintained. For all experiments examining chronic TNF-α exposure, Alzet osmotic pumps (Durect, Cupertino, CA) with a 7-day pumping capacity and infusion rate of 1 µl/h were used. Pumps were filled to capacity with 7.01 µg/ml TNF-α diluted in carrier (0.9% NaCl and 0.1% BSA). For apelin treatment, an intraperitoneal injection of 20 nmol/kg apelin-13 were performed for 10 min before the mice were sacrificed. For F13A treatment, 20 ug/mouse F13A were intraperitoneal injected for 30 min before the mice were sacrificed. Following induction of halothane general anesthesia, pumps were implanted into the intrascapular subcutaneous space. Incisions were closed with interrupted absorbable sutures. Liver tissues were removed surgically and frozen immediately in liquid nitrogen for further analysis.

All animal procedures were performed in accordance with the National Institutes of Health Animal Care and Use Guidelines. All animal protocols were approved by the Animal Ethics Committee at the Beijing Institute of Geriatrics.

### Cell culture

HepG2 cells (American Type Culture Collection) were cultured in minimum Eagle's medium (low glucose; Invitrogen) supplemented with 10% fetal bovine serum (Hyclone), 100units/ml penicillin (Invitrogen), and 0.1 mg/ml streptomycin (Invitrogen). Cells were maintained at 37°C with humidified air and CO2 (5%). Experiments on hepatocytes were performed in the presence of 10 ng/ml TNF-α for 24 h.

### Isolation of mouse primary hepatocytes

Male C57BL/6J mice (8-week-old) were provided from Peking University Health Science Center. Primary hepatocytes were isolated by a two-step collagenase perfusion (0.2 mg/ml type IV collagenase (Sigma) in Hanks balanced salt solution), as described previously [40], [41].The hepatocytes were collected by centrifugation at 800 rpm for 8 min. Immediately after harvesting, the cells were suspended in pre-warmed William's E medium (Sigma) supplemented with 10% fetal bovine serum, 20 ng/ml dexamethasone (Sigma), ITS (5 mg/l insulin, 5 mg/l trasferrin, 5 µg/l sodium selenate) (Sigma) and 10 µg/ml gentamicin (Invitrogen). Hepatocytes were plated in collagen-coated 25 cm2flask at 1×106 cells.

### Western blot analysis

Cell lysates (15–30 µg of protein) were separated by 10% SDS-PAGE, transferred to PVDF membrane (Millipore), blocked with 5% nonfat dry milk, and probed with antibodies at 4°C overnight. The blots were incubated with HRP-conjugated anti-IgG, followed by detection with ECL (Millipore). Antibodies against IRS1, phospho-IRS1, JNK, phospho-JNK, AKT, phospho-AKT, GSK and phospho-GSK were all purchased from Cell Signal Technology, USA. Antibody against apelin receptor APJ was obtained from Phoenix Pharmaceuticals, USA. To check insulin signaling molecules, cells were treated with 10 nmol/L insulin (Usbio) for 10 min before the protein was collected.

### Analysis of glycogen contents

Glycogen levels were measured in cells or liver tissues incubated for 3 h in the presence of 10 nmol/L insulin (Usbio), using a glycogen assay kit (Biovision).

### Statistical analysis

All values are represented as means ±SEM. of the indicated number of measurements. A one-way ANOVA test was used to determine significance, requiring p<0.05 for statistical significance.
