# Correction: Apelin Ameliorates TNF-α-Induced Reduction of Glycogen Synthesis in the Hepatocytes through G Protein-Coupled Receptor APJ

**DOI:** 10.1371/annotation/15dd6d58-5cea-4252-94a6-52778c6ee55e

**Published:** 2013-08-13

**Authors:** Jiaojiao Chu, Hangxiang Zhang, Xiuqing Huang, Yajun Lin, Tao Shen, Beidong Chen, Yong Man, Shu Wang, Jian Li

A fourth grant from the National Natural Science Foundation of China was incorrectly omitted from the Funding Statement. The number of the fourth grant is: 30900627. 

